# Intention to Engage in Winter Sport in Climate Change Affected Environments

**DOI:** 10.3389/fpubh.2020.598297

**Published:** 2020-12-18

**Authors:** Anika Frühauf, Martin Niedermeier, Martin Kopp

**Affiliations:** Department of Sport Science, University of Innsbruck, Innsbruck, Austria

**Keywords:** global warming, skiing, artificial snow, affective responses, behavior, theory of planned behavior, exercise

## Abstract

Exercise, including winter sport activities, shows positive effects on physical and mental health, with additional benefits when participating in natural environments. Winter sport activities are particularly vulnerable to climate change, since global warming will decrease the duration and amount of snow. In the context of climate change in alpine environments, little is known on the determinants of winter sport behavior. Thus, the following study primarily aimed at comparing the effect of being exposed to a climate change affected scenario (CCA) or to a climate change unaffected scenario (CCU) on the intention to engage in recreational winter sport activities. Secondly, we aimed to analyze the role of anticipated affective responses during exercising based on the Theory of Planned Behavior (TPB). An experimental cross-sectional web-based study design was used. Participants were randomly allocated to pictures of either CCA or CCU. TPB variables and affective responses with regard to the displayed scenarios were assessed. Statistical analyses included Mann-Whitney-*U* Tests, linear regression, and mediation analyses. Significant group differences were seen in all TPB variables, *p* < 0.038; −0.13 < *r* < −0.30, as well as in affective responses, *p* < 0.001; −0.24 < *r* < −0.85. Lower intention to engage in winter sport activities and lower anticipated affective valence during exercising was found in CCA compared to CCU. Attitude toward winter sport was significantly positively associated with intention to engage in winter sport, beta = 0.66, *p* < 0.001. The effect of group allocation on attitude was mediated by anticipated affective valence, indirect effect = 0.37, *p* < 0.001. Intention to engage in recreational winter sport activities was lower in participants exposed to the climate change affected winter sport scenario. Since affective valence seems to influence attitude and consequently intention to exercise, the role of non-cognitive variables with regard to climate change related exposure should be considered in future studies. Therefore, winter sport resorts may consider altered winter sport behaviors due to the consequences of climate change as well as the importance of providing an optimal framework to enhance affective valence of their guests in order to mitigate potential changes in winter sports behavior.

## Introduction

Physical inactivity is a major public health issue causing 9% of premature mortality which is equivalent to more than 5.3 million deaths in 2008 worldwide (1). Conversely, participating in regular physical activity is important to improve and maintain physical and mental health (2). Greater mental health benefits were shown when exercising in natural outdoor environments, along with a higher probability to change exercise behavior compared to exercising indoors (3–5). Demographically, higher amounts of natural space in the living area seem to be associated with less physical and mental disorders (6). However, ongoing urbanization leads to a reduction and loss of natural spaces (6). Natural environments can provide a venue and motivation to engage in exercise with additional mental health effects compared to exercising indoor (6).

However, exercising in natural environments is not only affected by urbanization but also by climate change. Alpine environments are especially prone to climate change vulnerability (7), where glacier retreat, less snowfall at lower altitudes and massive changes in the snow line were reported (8, 9). Large decreases of amount and duration of snow will be seen below 1,500–2,000 m altitude and snow amount will also decline at altitudes of 2,000 m above sea level within the next decades (8). This impact is accompanied with various severe consequences for people, such as water resource management, risk assessments, economic changes or tourism (9, 10). Based on economic cost models, climate change will affect the ski industry and skiing tourism negatively within the 21st century (11).

In addition, exercise behavior in alpine environments might change in the next 30 years due to climate change, especially for sports performed outdoors (12). Considering that six out of 10 of the most favorite sports in Austria are only possible to perform outdoors (e.g., alpine skiing, snowboarding, mountain hiking, or mountain biking). Together with a regional above average participation in winter sport activities (13), due to effects of climate change on exercise behavior are believed to be large in Austria. Given the importance of outdoor exercise and previous findings of additional physical and mental health benefits of exercising in alpine environments in various populations (5, 14–18), it seems important to analyze determinants of exercise behavior in the context of climate change.

Behavioral theories, such as the Theory of Planned Behavior [TPB; (19)] have commonly been used in the development of health related intervention strategies to evaluate a certain predicted future behavior (20, 21). TPB is also used in the context of exercise behavior and provides important determinants of intention to exercise: According to TPB, behavior (e.g., engaging in exercise) is determined to a large amount by behavioral intention (i.e., readiness to engage in exercise). Behavioral intention is based on subjective norm, attitude, and perceived behavioral control (22). Attitude and subjective norm are two social cognitive variables which influence the effect of intention toward behavior (23). Attitude represents an assessment of personal beliefs toward the target behavior. Subjective norm evaluates whether significant others want the person to engage in the target behavior (23). Attitude and social norm have been shown to influence both behavioral intention toward climate change action and behavioral intention to exercise (23–25).

TPB contains cognitive-oriented variables, which are not the only determinants of human behavior. In the exercise context, non-cognitive, affective processes play also an important role for behavior. Positive affective responses during exercise were shown to be associated with a higher level of future physical activity (26, 27). Although TPB allows adding additional variables to the model (26, 28), where to integrate affective responses to the TPB model is unclear. Affective responses during exercise may not influence subjective norm, but affective responses were predictive of attitudes and/or they might directly affect the intention to exercise (29). In the climate change context, being exposed to climate change scenarios provided by watching pictures have been used to trigger affective responses and consequently potentially change behavior (30). However, only positive pictures of climate change (such as solar panels) were shown to influence perceived behavioral control (31). Pictures showing global warming effects is used as a method to change threat appraisal of climate change and to engage people in climate-friendly actions (32). This might also have an effect on exercise behavior, especially when the exercise is conducted in environments sensitive to climate change. An Australian study revealed that with low natural snow scenarios, intention to ski regularly decreased from 70% in 1997 to 30% in 2007 (33).

Following these considerations, the goals of the consecutive study were as follows: We first aimed to analyze the effect of exposure to a climate change affected scenario on the intention to exercise in recreational winter sport activities compared to a climate change unaffected scenario. Attitude, subjective norm, and anticipated affective responses during exercising in the different scenarios were also compared. Secondly, we aimed to analyze the role of anticipated affective responses during exercising in the context of TPB.

## Materials and Methods

### Sample and Design

The data was collected via web-based questionnaire in a cross-sectional design. The questionnaire was distributed by University of Innsbruck's Email announcement and was received by mostly students. Due to local rights, it is possible to unsubscribe from the Email announcements, which does not allow giving an exact number of recipients. The Email announcement solely consisted of information and not mentioning the study goals but asking for motivation in recreational winter sport activities. No information about climate change was added. No incentives were provided for participating. The study procedure was approved by the Board for Ethical Questions in Science of the University of Innsbruck in accordance with the Declaration of Helsinki (No. 25/2016, date: 18.07.2016).

### Procedure and Randomization

**Figure 1:**
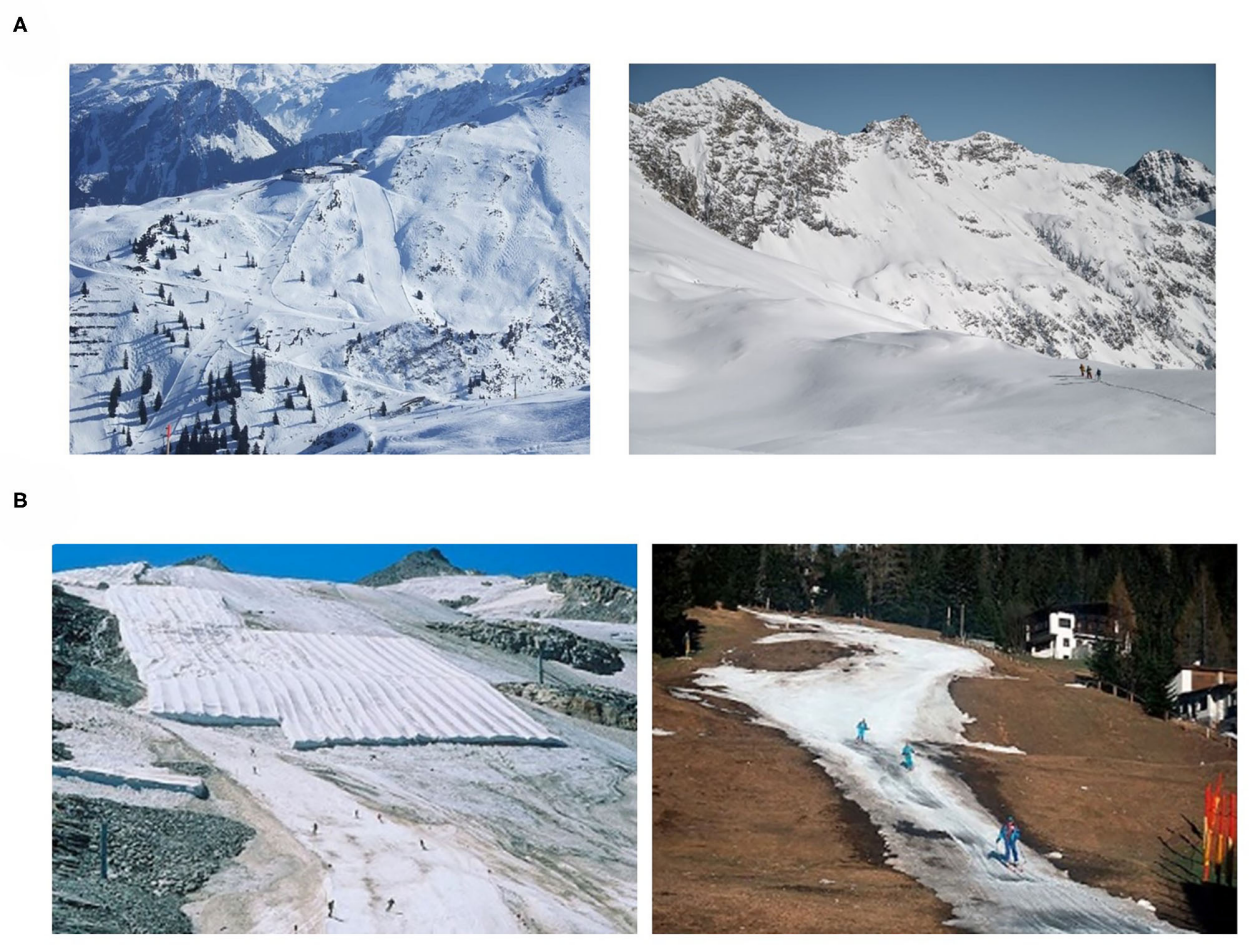
Climate change unaffected scenario (**A**, upper row) and climate change affected scenario (**B**, lower row). Pictures B by courtesy of “Gesellschaft für ökologische Forschung.”

Information about socio-demographic characteristics, control variables, affective responses, TBP variables, and future winter sport engagement were collected with a total of 34 questions. Time to complete the questionnaire was approximately 10 min. Once participants started the questionnaire, the first page on sociodemographic and control variables appeared prior to randomization and was identical for both groups. With the start of second page, participants were randomized (simple randomization) to one of two groups: (1) A climate change affected scenario was provided to one group (CCA group) and (2) a climate change unaffected scenario to the other group (CCU group). The scenarios were displayed by two pictures each (Figure 1). The pictures were placed at the top of the page asking participants to look closely at the scenarios and answer the following questions about a possible engagement in winter sport activities in the corresponding scenario.

### Measurements

#### TPB Variables Assessment and Future Winter Sport Engagement

Behavioral intention as well as attitude toward behavior and subjective norm were assessed following the TPB guidelines by Ajzen (34). Since this study was not primarily aimed at maintaining a change in health behavior, the dimension of “perceived behavior control” was not considered; additionally picture exposure to climate change effects (such as flooding) did not change “perceived behavioral control” (31), this study did not assess “perceived behavioral control.” All questions were answered on a seven-point Likert scale with reference to winter sport participation in the displayed scenario with attention to the upcoming winter. Intention to engage in winter sport was assessed by using three items asking about intention to engage regularly in winter sport activities (e.g., “I intend to participate regularly in winter sport activities in the upcoming winter.”). Cronbach's alpha for intention to engage in winter sport in the present sample was 0.89. Attitude toward winter sport was assessed using five items with two opposing attributes as anchors at the extreme sides (e.g., harmful/beneficial, pleasant/unpleasant, or good/bad, Cronbach's alpha = 0.89). Subjective norm was evaluated by applying four items about other people's opinion on the exercise behavior [e.g., “Most people who are important to me think that I should participate in winter sport activities regularly (at least once a week).”]. Cronbach's alpha for subjective norm was 0.79. After recoding the negatively formulated items, the mean of all items belonging to each TPB variable was calculated resulting in values ranging from 1 to 7 with higher numbers presenting higher intention, subjective norm, or attitude.

As an additional variable not used in the TPB a single-item question by Pickering et al. (33) on future winter sport engagement in the case of fewer snowfalls in the next five winters was asked. The question contained four response options ranging from “Quit winter sport,” “Conduct winter sport further away,” “Conduct winter sport less often than now,” and “Conduct winter sport in the same frequency as now.”

#### Affective Responses

Affective responses were assessed immediately after first exposure to the scenarios. Two single-items measures based on the Circumplex Model were used to assess two dimensional affective states affective valence and perceived activation (35). Affective valence was assessed by the Feeling Scale [FS; (36)]. The FS provides anchors at zero (“Neutral”) and at all odd integers, ranging from “Very good” (+5) to “Very bad” (−5). Convergent validity for the FS has been established previously (36, 37). Perceived activation was assessed by the Felt Arousal Scale [FAS; (38)]. This rating scale ranges from 1 (“low arousal”) to 6 (“high arousal”). The FAS has been used in previous studies, demonstrating convergent validity with other measures of perceived activation (37). FS and FAS have been validated in German language recently (39). Both FS and FAS were referring to the anticipated affect when engaging in winter sport in the displayed scenarios.

#### Control Variables

Regular participation in winter sport activities and specification of winter sport activities were assessed using nine items with possible answers ranging from 1 (never) to 7 (regularly). Non-winter sport specific physical activity level was assessed with two questions asking about physical activity during leisure time and at work (40). It allows to estimate a physical activity level indicator ranging from 1.4 (low physical activity level) to 2.3 (high physical activity level). Additionally, energy expenditure (expressed in MJ) is estimated taking into account the physical activity level, weight status, age group, and sex. The questionnaire was validated against the doubly labeled water method and showed acceptable characteristics to be used in epidemiological studies (40). Risk-perception of climate change was assessed using a short version of the risk-perception scale with three items and four response options each (41). The mean of the three items was calculated resulting in values ranging from 1 (low perceived risk of climate change) to 4 (high perceived risk of climate change).

### Statistical Analyses

Statistical analyses consisted of tests on group differences, linear regression, and mediation analyses. Group differences between CCA and CCU group were analyzed with the Mann-Whitney *U* Test due to non-normality of data (tested by the Shapiro-Wilk test for each subgroup) using SPSS v. 26 (IBM, New York, NY, USA). TPB variables (intention to engage in winter sport, attitude toward winter sport, and subjective norm) and anticipated affective responses during winter sport (affective valence and perceived activation) were used as the outcomes. Rosenthal's *r* was calculated as an effect size in the group difference analysis with the conventions small (0.1), medium (0.3), large (0.5) (42). For evaluating group differences in percentages of future winter sport engagement in case of less snowfall, Pearson's χ^2^ test was used.

Linear regression and mediation analyses were performed using jamovi v. 1.2 (43). Mediation analysis was done along the module jAMM (44) with group allocation presenting the predictor, in addition affective valence during winter sport as the mediator and attitude toward winter sport as the outcome. Affective valence was regarded as a mediating variable in the presence of a significant indirect effect of group allocation on attitude toward winter sport. Given previous findings that affective responses were predictive of attitude and not predictive of subjective norm, attitude was chosen as the outcome variable (29). Bias-corrected bootstrap 95% confidence intervals (95% BC CI) were calculated on the basis of 1,000 samples for the unstandardized regression coefficient of the indirect effect. In the regression analysis, the outcome intention to engage in winter sport was modeled by the predictors of the TPB (attitude and subjective norm), affective valence, and group allocation. *P*-values < 0.05 were considered as significant. Unless otherwise stated, values represent mean (SD), Median (interquartile range), and relative (absolute) frequencies.

## Results

### Sample Characteristics

Total sample size was *n* = 214 participants, of which 47.3% (*n* = 101) were allocated to the climate change affected scenario and 52.8% (*n* = 113) were allocated to the climate change unaffected scenario. Both CCA and CCU groups were relatively similar in regards to demographic aspects, physical activity, and winter sport activities (Table 1). The majority of the sample was regularly engaging in slope and freeride skiing/snowboarding. Risk-perception index of climate change was similar for both groups (mean of the total sample 3.1 on the scale of 1 to 4).

**Table 1:** Data on demographic aspects, physical activity, risk-perception of climate change, and winter sport activities of the total sample and by group.

	**Total sample (*****n*** **=** **214)**		**Climate change unaffected scenario (*****n*** **=** **113)**		**Climate change affected scenario (*****n*** **=** **101)**	
	**Mean**	**(SD)**	**Mean**	**(SD)**	**Mean**	**(SD)**
Age (years)	23.5	(4.7)	23.8	(5.5)	23.0	(3.5)
Body mass index (kg/m^2^)^a^	22.3	(2.6)	22.1	(2.6)	22.5	(2.6)
Physical activity level^a^	1.8	(0.2)	1.9	(0.2)	1.8	(0.2)
Energy expenditure (MJ)^a^	11.8	(2.6)	11.6	(2.6)	12.0	(2.5)
Risk-perception of climate change(1: low, 4: high)	3.1	(0.5)	3.1	(0.6)	3.2	(0.5)
Frequency of winter sport activities (n/season)^a^	12.1	(10.2)	13.7	(11.5)	10.4	(8.1)
	**%**	**(*****n*****)**	**%**	**(*****n*****)**	**%**	**(*****n*****)**
Sex
Female	64.5	(138)	69.0	(78)	59.4	(60)
Male	35.5	(76)	31.0	(35)	40.6	(41)
Regular winter sport activity (>4 out of 7)^a^						
Slope skiing/snowboarding	84.0	(178)	84.8	(95)	83.0	(83)
Freeride skiing/snowboarding	52.4	(109)	58.6	(65)	45.4	(44)
Ski/snowboard touring	43.0	(89)	43.1	(47)	42.9	(42)
Toboganing	16.3	(34)	13.5	(15)	19.6	(19)
Winter hiking	14.6	(30)	11.8	(13)	17.7	(17)
Ice climbing	1.5	(3)	1.9	(2)	1.1	(1)

a *missing data: n < 9*.

### Group Differences

All TPB variables were significantly different between groups (Table 2). Intention to engage in winter sport, attitude toward winter sport, and subjective norm were rated significantly lower in the CCA group, who watched the climate change affected scenario, compared to the CCU group, who watched the climate change unaffected scenario. Effect size of group differences was largest for attitude followed by intention and subjective norm. Similarly, the CCA group rated both affective valence and perceived activation during winter sport significantly lower compared to the CCU group.

**Table 2:** Theory of planned behavior variables, affective responses, and future winter sport activity engagement by group (*n* = 214).

	**Climate change unaffected scenario (*****n*** **=** **113)**				**Climate change affected scenario (*****n*** **=** **101)**						
	**Mean**	**(SD)**	**Med**	**(IQR)**	**Mean**	**(SD)**	**Med**	**(IQR)**	***z***	***p***	***r***
Theory of Planned Behavior variables (1: low, 7: high)											
Intention to engage in winter sport	6.7	(0.8)	7.0	(7.0–7.0)	6.3	(1.3)	7.0	(6.3–7.0)	−3.66	** <0.001**	–**0.25**
Attitude toward winter sport	6.6	(0.8)	7.0	(6.3–7.0)	5.9	(1.3)	6.2	(5.4–7.0)	−4.30	** <0.001**	–**0.29**
Subjective norm^a^	5.1	(1.5)	5.3	(4.0–6.3)	4.6	(1.6)	4.7	(3.7–6.0)	−2.08	**0.038**	–**0.14**
Affective responses during winter sport											
Affective valence	4.1	(1.7)	5.0	(3.5–5.0)	−2.8	(1.9)	−3.0	(−4.0−1.0)	−12.35	** <0.001**	–**0.84**
Perceived activation^a^	4.5	(1.4)	5.0	(3.0–6.0)	3.6	(1.8)	4.0	(2.0–5.0)	−3.71	** <0.001**	–**0.25**

a *missing data: n < 4, Med, Median; IQR, interquartile range. Bold values indicate significant group differences*.

**Figure 2:**
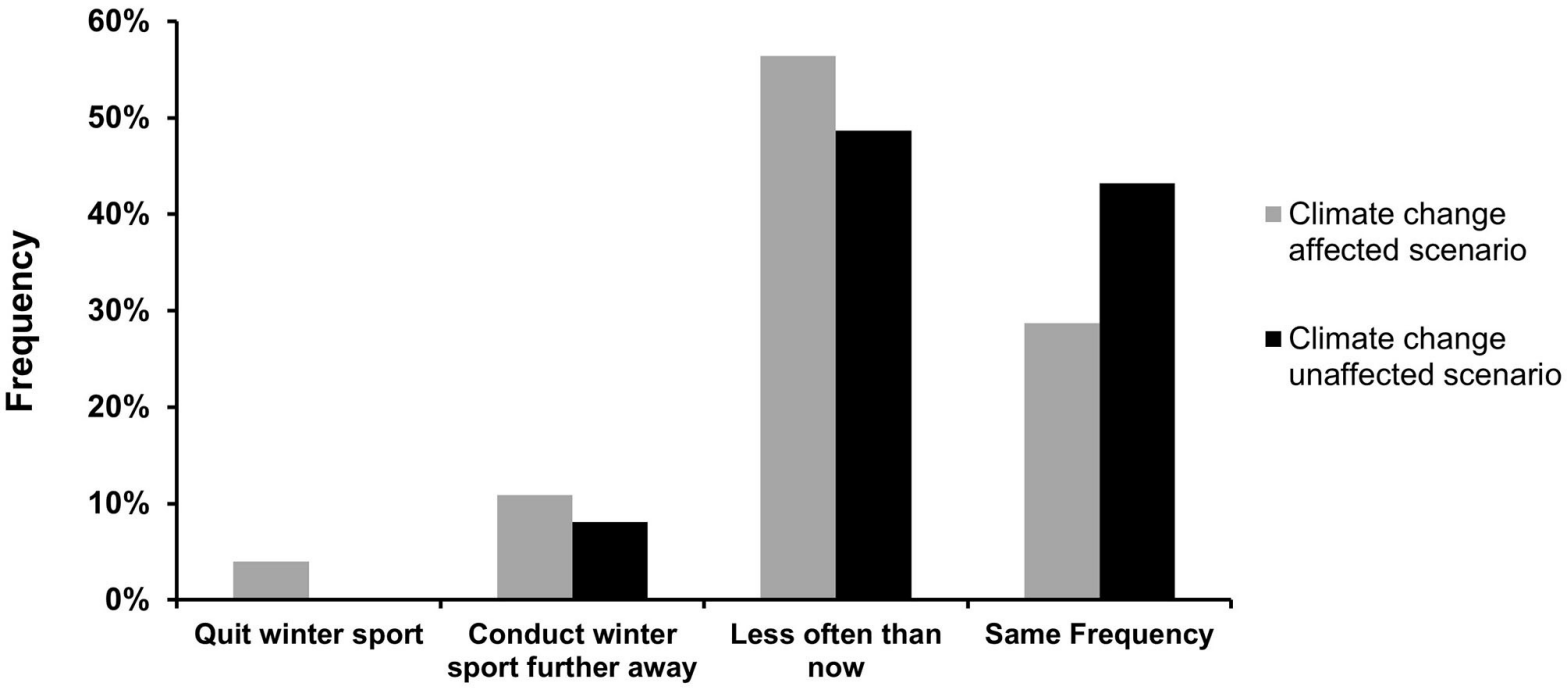
Future winter sport engagement by group. Percentages were significantly different, *p* = 0.036.

The frequencies in the categories of future winter sport engagement in case of less snowfall was significantly different between groups, χ^2^ (3, *N* = 212) = 8.52, *p* = 0.036. The largest discrepancy between groups was in the category “same frequency” (Figure 2). Out of the CCA group, 28.7% responded to engage in their winter sport in the same frequency in case of less snowfall. In the CCU group, this percentage was 43.2%.

### Regression and Mediation Analyses

**Figure 3:**
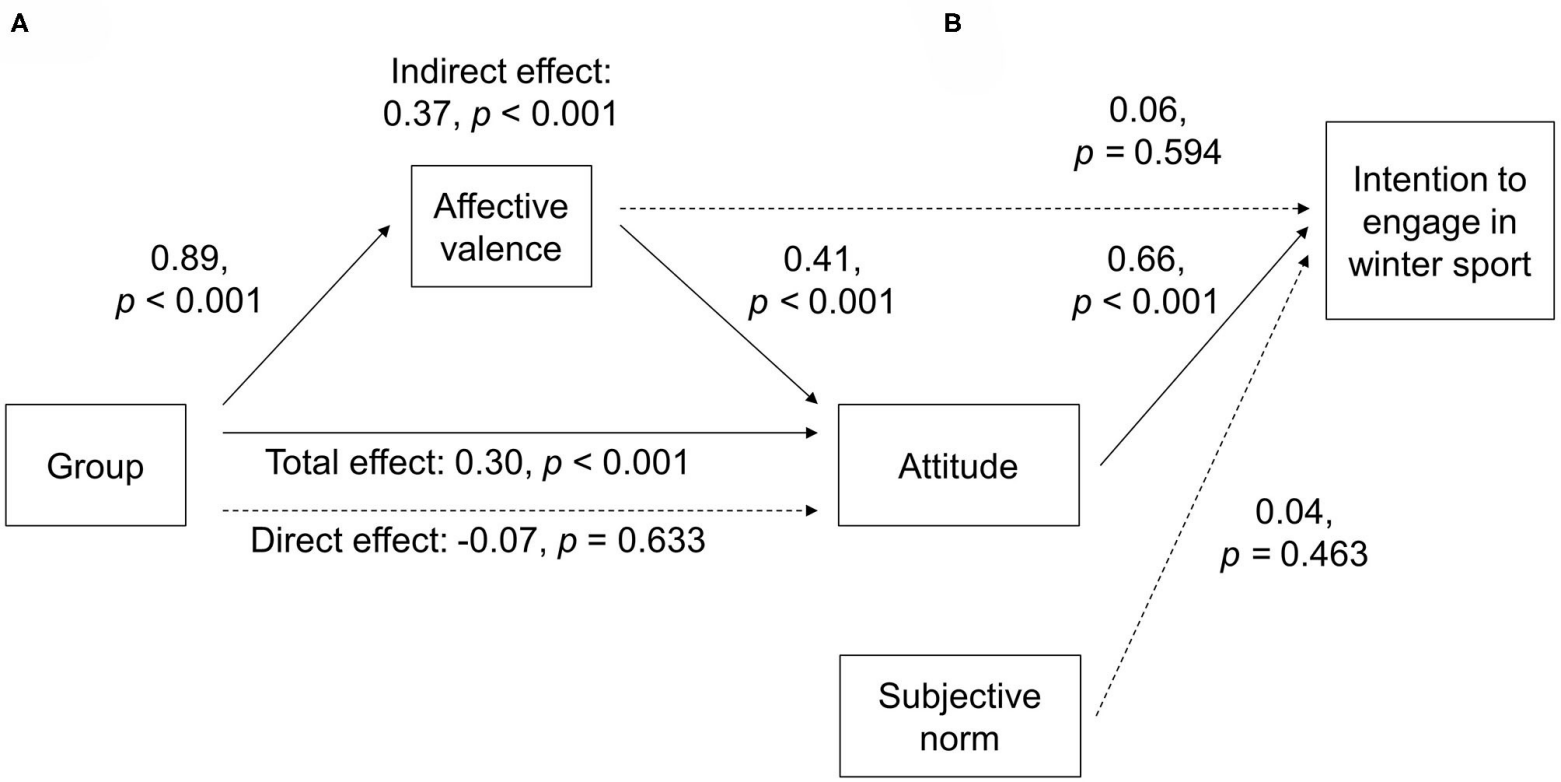
Model of attitude as an outcome of group allocation mediated by affective valence (**A**, mediation analysis) and model of intention to engage in winter sports as an outcome of affective valence, attitude, subjective norm, and group allocation (**B**, regression analysis). Path coefficients show standardized regression coefficients. Solid lines indicate statistically significant coefficients; dashed lines indicate non-significant coefficients.

There was a significant indirect effect of group allocation on attitude toward winter sport through affective valence (Figure 3A), unstandardized regression coefficient = 0.83, 95% BC CI: 0.41–1.34, *p* < 0.001. The analysis indicates that the effect of group on attitude was mediated by anticipated affective valence.

Attitude toward winter sport was significantly positively associated with intention to engage in winter sport (Figure 3B). Both the effect of subjective norm and affective valence on intention to engage in winter sport was not significant. Similarly, the direct path of group allocation on intention to engage in winter sport was not significant [standardized regression coefficient = −0.13, *p* = 0.561 (not shown in Figure 3)].

## Discussion

The primary aim of the present study was to analyze the effect of exposure to a climate change affected scenario on the intention to engage in recreational winter sport activities compared to a climate change unaffected scenario. Further cognitive and non-cognitive variables were also compared. Main results include significantly lower intention to engage in recreational winter sport, as well as attitude, subjective norm, anticipated affective responses during exercising and future winter sport engagement when exposed to a climate change affected scenario. Furthermore, anticipated affective valence during winter sport in the scenario was not significantly associated with intention to exercise. Instead, affective valence mediated the effect of being exposed to a climate change affected scenario on attitude.

### Climate Change Affected Scenario and Intention

The present results suggest that people report lower intention to engage in winter sport activities when they are exposed to a climate change affected scenario. Intention to exercise was repeatedly shown to be a highly important predictor of actual exercise behavior (23). Therefore, we assume that people are less likely to participate in winter sport activities when they are exposed to climate change affected scenarios. Climate change affected scenarios are expected to appear more commonly given the visible consequences connected with climate change in alpine environments, e.g., glacier retreat or less snowfall at lower altitudes (8, 9). Therefore, our findings have important implications both on the exercise behavior of people in alpine environments and on tourist industry as well as economical aspects of these regions.

In regards to tourist industry and economical aspects, especially ski resorts at lower altitudes have to use artificial snow to make ski slopes skiable (45). In the previous years, around 20% of sales from ski resorts were needed for artificial snow production (45). Further artificial snow making will result in higher costs and need for additional resources (e.g., water or energy) (46). However, our climate change affected scenario included a picture of artificial snow in an otherwise green area. If tourists experience climate change affected scenarios in their vacations and show a lower intention to engage in winter sport activities, it is less likely that they return to the ski resorts in the future. This is hypothesized since snow and winter experience are the two highest ranked destination choice determinants of winter sport tourists (47). Therefore, ski resorts might face a lower number of winter sport participants, when climate change affected scenarios are more frequently seen in addition to higher maintenance costs (11). Few studies have assessed intention to ski in case of lack of snow in the upcoming years and received a considerable amount of participants who would either stop skiing or ski less (33, 47, 48). In a study conducted 20 years ago in Europe, 40% answered to ski less often or give up skiing (48). The same question was asked in the present study and 54.2% of the total sample answered to engage in winter sport less often or quit winter sport. Although the question referred to less snowfall in the upcoming seasons and was unrelated to the displayed scenarios, group differences were seen with a lower amount of people in CCA reporting to engage in their winter sport in the same frequency than now. Since economic cost models have largely neglected the aspect of skiers' intention to exercise in regard to climate change (11), future model calculations should take an altered demand from winter sport participants into account.

From a public health perspective, it is important that the exercise level of people living in alpine environments do not decline, given the health benefits of exercising in alpine environments (5, 16–18). However, the present study showed that the intention to engage in winter sport activities was lower when exposed to a climate change affected scenario. It is evident that measures against climate change on an individual level are recommended to avoid a lower intention in winter sport activities. Protection Motivation Theory suggests, that people are more likely to perform a precautionary behavior if the personal threat appraisal is high (41, 49). This is also favorable in the light of further (global) negative consequences (41). Thus, pictures of climate change affected scenarios might be used as a trigger for the assessment of climate change behavior in future studies. However, since climate change is a global phenomenon and is likely to continue in the future, other solutions for a potential decline of exercise level in a climate change affected scenario have to be found. Our analyses indicated that intention to engage in winter sport activities is not directly influenced by the exposure to the scenarios. Instead, attitude toward winter sport was at least partially mediated by anticipated affective responses during exercising. Therefore, it seems important for preventive measures against a potential decline in these specific snow-associated exercise behaviors to focus on the affective valence in winter sport [i.e., by providing stimuli for positive affect, e.g., nature experience or reducing costs for ski passes (47)]. Furthermore, other forms of exercise (e.g., mountain biking or mountain hiking) that are still possible to be conducted in a climate change affected environment, might be promoted as all-year round activities (5, 46). In this context, it should be noted that besides a reduction of physical activity in extreme temperatures (both hot and cold), model calculations in the US population revealed an *increase* in net recreational physical activity through warmer winters (50). Therefore, climate change might be even seen as a possibility for higher exercise levels in other forms of exercise than winter sport. Although it is questionable how these results based on the US population are transferable to the Austrian population due to a different exercise behavior (51).

### Affective Valence in the Context of TPB Variables

The second aim of this study was to analyze the role of anticipated affective responses during engaging in winter sport activities in the context of TPB. Given previous findings on the importance of affective valence in the prediction of future exercise behavior (26, 27), and the strong relation between intention to exercise and future exercise (29), we expected a larger influence of affective valence in the intention to engage in winter sport activities. However, affective valence was not significantly associated with intention to engage in winter sport activities in the present sample. Attitudes toward winter sport were significantly (positively) associated with intention to engage in winter sport, which was similarly found in previous studies (23, 29). Attitudes in turn, were significantly associated with anticipated affective responses during engaging in winter sport. Therefore, affective valence seems to be important to consider when focusing on the attitude toward an exercise behavior (opposed to directly on the intention). Attitude had the strongest influence on exercise intention, whereas subjective norm only marginally influenced intention (23). In the present study, subjective norm failed to reach significance in the effect of intention to engage in winter sport. When designing the study, subjective norm was seen important in the context of climate change since subjective norm was an important predictor of eco-friendly related behavior (52) and was suggested to be triggered by climate change scenarios. However, as shown in other exercise research, subjective norm might not be a key predictor of intention to exercise (23).

With regard to engagement in climate change, the potential on affective responses of pictures was acknowledged previously: Ockwell et al. (53) specified three key-components, namely the cognitive component of understanding and knowledge, the affective component and the behavioral component. Climate change pictures can trigger emotions and have a stronger effect on the emotional system if they rely on local scenarios; as this was the case in the present study (54). As seen in risk-research, when cognitive and emotional (affective) assessments diverge, the emotional assessment can drive behavior (55). Given the large effect size in affective valence found in the present study, we cautiously transfer the potential of pictures on affective responses for the exercise intention context. Although pictures with CCA scenarios did not directly change intention or attitude toward winter sport activities, they have the potential to change attitude toward winter sport engagement through changing anticipated affective valence during winter sport. Thus, the non-cognitive variables (i.e., affective valence) should be considered when trying to engage people in exercise behavior (26).

### Limitations and Strengths

When interpreting the results of the present study, the following limiting factors have to be considered. Firstly, we applied a cross-sectional study design and only assessed intention without controlling for actual behavior. Secondly, we acknowledge a potential selection bias. The present sample was relatively young, showed a relatively high risk-perception index (41), and a high physical activity level predominantly engaging in slope skiing/snowboarding. It is unclear, how these results are transferable to people being less sensitive to risk perception of climate change, being less physically active (56), or engaging in other winter sports than slope skiing/snowboarding.

Albeit, to the best of our knowledge, the present study is the first randomized study, which assesses effects of climate change affected and unaffected scenarios on cognitive and affective determinants of exercise behavior. Reliable randomization was concluded based on a similar risk-perception score and similar physical activity behavior between the groups.

## Conclusion

Intention to participate in recreational winter sport activities was lower in participants exposed to the climate change affected winter sport scenario. The displayed scenarios influenced affective valence, which was a determinant on attitude and thus in consequence on intention to engage in winter sport activities. This research has mainly two practical implications, one for economists and one for exercise psychologists: (a) winter sport resorts should consider an altered intention to engage in winter sport activities (and consequently in potential behavior) in the presence of climate change affected scenarios; (b) affective valence does not seem to influence intention to engage in winter sports directly. Instead, affective valence seems important to influence attitudes and seems to be a mediating variable in the effect of being exposed to climate change affected scenarios on attitude. Thus, the role of non-cognitive variables with regard to climate change related picture exposure should be considered in future studies and might be interesting for climate change communication usage. Future studies might integrate an assessment of actual winter sport behavior in winters with little snow as well as a possible change of winter sport toward other alpine sport activities.

## Data Availability Statement

The raw data supporting the conclusions of this article will be made available by the authors, without undue reservation.

## Ethics Statement

The studies involving human participants were reviewed and approved by Board for Ethical Questions in Science of the University of Innsbruck. Written informed consent for participation was not required for this study in accordance with the national legislation and the institutional requirements.

## Author Contributions

AF, MN, and MK contributed to conception and design of the study. AF and MN collected data. MN analyzed the data and wrote the results. AF wrote a first draft of the manuscript. All authors contributed to the manuscript and approved the submitted version.

## Conflict of Interest

The authors declare that the research was conducted in the absence of any commercial or financial relationships that could be construed as a potential conflict of interest.
